# Pathology and Therapeutics of the Domestic Animals

**Published:** 1895-01

**Authors:** 


### REVIEWS.

Friedberger and Frohner's *Pathology and Therapeutics of the Domestic Animals*, Volume 1. Translation by Prof. W. L. Zuill, of Philadelphia. No more welcome volume has come to the English-speaking and reading veterinarian than this admirable work of so much everyday value to the general practitioner in veterinary medicine. Elegantly printed, with clear, distinct type, bound in cloth, it bears evidence of the highest character of the printer's art. There are no higher authorities the world over than those whose labors produced this valuable contribution for their fellow-countrymen, and no better work could have been offered to those who are dependent on English literature for assistance and advancement than this splendid contribution of Prof. Zuill. No veterinary or comparative science library can be complete without this work, and none will be more appreciated We bespeak for it a wide circulation, a generous reception, and the grateful appreciation of all for the labor and expense that have placed it within the reach of so many and among those where there existed so great a need for such a work.

